# Decreased expression of cell proliferation-related genes in clonally derived skin fibroblasts from children with Silver-Russell syndrome is independent of the degree of 11p15 *ICR1* hypomethylation

**DOI:** 10.1186/s13148-014-0038-0

**Published:** 2015-01-22

**Authors:** Doreen Heckmann, Christina Urban, Karin Weber, Kai Kannenberg, Gerhard Binder

**Affiliations:** Pediatric Endocrinology, University Children’s Hospital Tuebingen, Hoppe-Seyler-Straße 1, 72076 Tuebingen, Germany

**Keywords:** Silver-Russell syndrome, IGF2, H19, Fibroblasts, Methylation

## Abstract

**Background:**

The *in vitro* analysis of the hypomethylation of *imprinting control region 1 (ICR1)* within the *IGF2/H19* locus is challenged by the mosaic distribution of the epimutation in tissues from children with Silver-Russell syndrome (SRS). To exclude mosaicism, clonal cultures of skin fibroblasts from four children with SRS and three controls were analyzed. Cell proliferation, IGF-II secretion, and *IGF2* and *H19* expression were measured, and a microarray expression analysis was performed.

**Results:**

Single-cell expansion established severely *ICR1* hypomethylated clones (SRShypo) and normomethylated clones (SRSnormo) from the patients and controls (Cnormo). *IGF2* expression was below the detection limit of the quantitative real-time PCR (qRT-PCR) assay, whereas *H19* expression was detectable, without differences between fibroblast clones. Cell count-related IGF-II release was comparable in SRShypo and Cnormo supernatants. Cell proliferation was diminished in SRShypo compared to Cnormo (*p* = 0.035). The microarray analysis revealed gene expression changes in SRS clones, predicting a decrease in cell proliferation and a delay in mitosis.

**Conclusions:**

The analysis of severely *ICR1* hypomethylated clonal fibroblasts did not reveal functional differences compared to normomethylated clones with respect to *IGF2* and *H19* expression. A difference compared to the clones from healthy individuals was present in the form of a lower proliferation rate, presumably due to impaired cell cycle progression.

**Electronic supplementary material:**

The online version of this article (doi:10.1186/s13148-014-0038-0) contains supplementary material, which is available to authorized users.

## Background

Silver-Russell syndrome (SRS, OMIM 180860) is a sporadic, clinically, and genetically heterogeneous disorder characterized by severe intrauterine and postnatal growth failure, a typical triangular face, asymmetric growth of the body, relative macrocephaly, and underweight [1]. SRS is basically associated with two different epigenetic defects. Approximately every tenth SRS patient inherits both copies of chromosome 7 from the mother (maternal uniparental disomy 7, matUPD7), though the biological mechanism by which matUPD7 causes the phenotype remains unclear. The other approximately half of SRS patients carry a hypomethylation of the paternal imprinting center region 1 (*ICR1*) on chromosome 11p15; these patients exert the most severe phenotype. The hypomethylation is of different severity in individual patients, individual tissues, and even individual cells of the same patient [2], indicating a mosaic distribution. Additionally, an association with the hypomethylation of other imprinted loci, called multilocus imprinting defect, has been observed in approximately 10% of SRS patients with *ICR1* hypomethylation on 11p15 [3-6].

The expression of the genes *IGF2* and *H19* is allele-specific due to imprinting. *ICR1* on 11p15 controls the expression of the imprinted genes *IGF2* and *H19*. Physiologically, *H19* is only expressed from the maternal unmethylated allele, whereas *IGF2* is only expressed from the fully methylated paternal allele. It is thought that hypomethylation of the paternal *ICR1* allele might result in the reduced production of the important fetal growth factor IGF-II [7]. In contrast, hypermethylation on the maternal allele in Beckwith-Wiedemann syndrome might result in increased IGF-II production and a predisposition toward tumor growth [8]. *H19* encodes a capped, spliced, and polyadenylated noncoding 2.3-kb RNA with unclear function [9,10]. *H19* is highly expressed from the early stages of embryogenesis to fetal life in many organs including the fetal adrenal, liver, and placenta tissues but is nearly completely downregulated postnatally [11].

IGF-II is the essential growth factor for intrauterine growth. *IGF2* knockout mice showed impaired growth kinetics and nearly 36% loss of weight in comparison to wild-type animals [12]. Children with SRS and *ICR* hypomethylation had normal or even high serum IGF-II levels [13], which may be explained by the biallelic hepatic expression of IGF-II after birth.

Two miRNAs are expressed from the *IGF2/H19* locus: *miR-675* embedded within the first exon of *H19* [14-17] and *miR-483* derived from the second intron of *IGF2* [18]. *miR-483* was found to be co-expressed with *IGF2* in tumors [19] and was identified as a possible regulator of *IGF1* expression in human natural killer cells [20]. In keratinocytes, the accumulation of *miR-483-3p* blocks cell cycle progression via the direct repression of *CDC25A* and the subsequent disassembly of CCND-CDK4/6 [21]. It has been hypothesized that the two miRNAs expressed from the *IGF2/H19* locus may contribute to the etiology of SRS.

The first study on *ICR1* hypomethylation in SRS reported a decreased *IGF2* expression in skin fibroblasts from SRS children with severe *ICR1* hypomethylation [7]. However, we recently did not observe a significant correlation between *ICR1* hypomethylation and *IGF2/H19* expression in skin fibroblasts from children with SRS [22], though the *ICR1* hypomethylation of these skin fibroblasts was significantly milder than in the blood leukocytes of the same patients and milder than in the patients studied by Giquel et al. [7]. Furthermore, the body asymmetry of these children with SRS was not related to the degree of hypomethylation in the skin fibroblasts collected from the two differently growing arms.

Here, we expand our *in vitro* analysis of skin fibroblasts from patients with SRS to monoclonal cultures with severe *ICR1* hypomethylation. In addition to analyzing cell proliferation rates, we studied the expression of *IGF2*, *H19*, and miRNAs originating from the *IGF2/H19* locus in normo- and severely hypomethylated clones. In addition, we performed gene expression profiling by a microarray analysis and compared the results with healthy controls.

## Results

### Clone selection by methylation profiling

Single cells from skin-derived primary fibroblast cultures were picked and expanded in culture for establishing clonal fibroblast cultures from SRS patients and controls. The quantification of the degree of methylation of the CpG sites M1–M4 in *ICR1* by methylation-specific multiplex ligation-dependent probe amplification (MS-MLPA) enabled the selection of severely hypomethylated (methylation <10%, SRShypo) and normomethylated (methylation 30%–55%, SRSnormo) clones from the patients as well as of normomethylated clones from the controls (methylation 39%–45%, Cnormo) (Table 1). The methylation status of *ICR1* and *ICR2* at the *IGF2/H19* locus was found to be stable between passages 4 and 17 (data not shown). All experiments were performed at passages 12–18. The MS-MLPA results were confirmed by bisulfite sequencing (data not shown).

**Table 1:** **Analysis of the methylation at the imprinting center region 1 (** ***ICR1*** **) and** ***ICR2*** **on 11p15 in clonal fibroblasts**

		**ICR1 methylation (mean; M1–M4)**	**ICR1 methylation (single methylation sites)**				**ICR2 methylation**
			**M1**	**M2**	**M3**	**M4**	
SRShypo	S1sh_6	0 ± 0	0 ± 0	0 ± 0	1 ± 1	0 ± 0	50 ± 3
	S1sh_8	0 ± 0	0 ± 0	0 ± 0	1 ± 0	0 ± 0	52 ± 6
	S5lo_10	0 ± 0	1 ± 0	0 ± 0	1 ± 1	0 ± 0	45 ± 3
	S2lo_7	1 ± 1	0 ± 0	0 ± 0	1 ± 1	2 ± 2	42 ± 7
	S2lo_1	1 ± 2	0 ± 0	0 ± 0	5 ± 2	0 ± 0	48 ± 2
	S1sh_12	1 ± 2	0 ± 0	0 ± 0	5 ± 2	0 ± 0	55 ± 5
	S5sh_11	2 ± 2	0 ± 0	0 ± 0	3 ± 1	5 ± 5	51 ± 4
	S3lo_9	8 ± 10	1 ± 1	0 ± 0	3 ± 1	28 ± 1	33 ± 3
SRSnormo	S3sh_19	39 ± 10	26 ± 8	2 ± 1	53 ± 3	46 ± 0	49 ± 1
	S5lo_9	33 ± 11	13 ± 2	32 ± 8	46 ± 7	41 ± 2	51 ± 3
	S2sh_1	30 ± 16	29 ± 3	29 ± 1	45 ± 5	47 ± 0	45 ± 7
	S1sh_9	43 ± 17	35 ± 6	19 ± 2	60 ± 12	60 ± 0	52 ± 5
	S1lo_7	55 ± 16	40 ± 3	37 ± 2	55 ± 7	86 ± 8	46 ± 2
Cnormo	K3li_3	45 ± 11	27 ± 4	41 ± 4	50 ± 4	64 ± 1	53 ± 3
	K2li_17	44 ± 9	38 ± 2	41 ± 6	53 ± 2	54 ± 1	60 ± 3
	K3re_12	39 ± 6	28 ± 4	46 ± 1	39 ± 1	44 ± 2	61 ± 5
	K1li_5	44 ± 4	28 ± 2	45 ± 6	49 ± 1	43 ± 9	59 ± 6

Numbers represent percentages of methylation (100% = fully methylated) ± standard deviation measured by MS-MLPA (*n* ≥ 2). The following sequences were amplified: M1: 1975955 (start)–1976006 (end), M2: 1976099–1976159, M3: 1976269–1976321, M4: 1976583–1976631.

### Proliferation analysis

**Figure 1:**
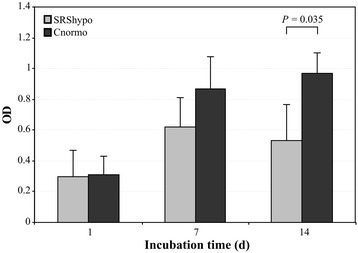
**Proliferation rates of SRShypo clones and Cnormo clones.** Fibroblast clones were cultured for 14 days (d) in standard medium. Proliferation of four different SRShypo clones (S1sh_12, S2lo_7, S2lo_1, S5sh_11) and three different Cnormo clones (K2li_17, K3li_3, K3re12) was measured using the MTT assay after 1, 7, and 14 days.

Cell proliferation during the 14 days of culture, as measured by the 3-(4,5-dimethylthiazol-2-yl)-2,5-diphenyl tetrazolium bromide (MTT) assay, was slightly lower in the SRShypo group (S1sh_12, S2lo_7, S2lo_1, S5sh_11) than in the Cnormo group (K2li_17, K3li_3, K3re_12) after 7 days and significantly lower after 14 days of culture (Figure 1) (*p* = 0.035). Cell proliferation slowed after 1 week of culture in the Cnormo clones and stagnated in the SRShypo clones.

### Expression of *IGF2* and *H19*

**Figure 2:**
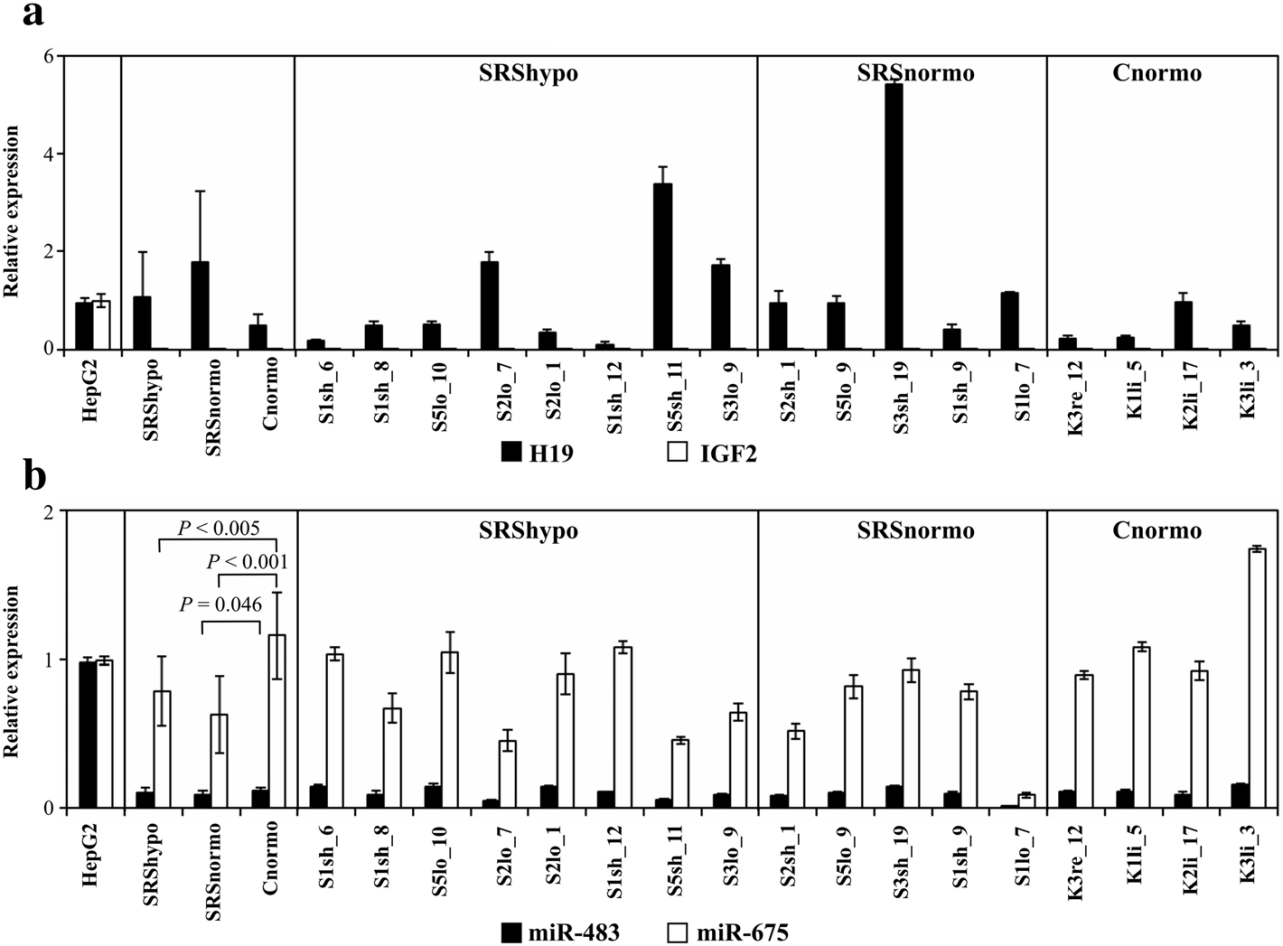
**Expressions of** ***IGF2*** **and** ***H19*** **and of** ***miR-483*** **and** ***miR-675.*** **(a)** Expression of *IGF2* and *H19* in fibroblast clones. qRT-PCR results were normalized to gene and miRNA expression in HepG2 cells. **(b)** Expression of *miR-483* and *miR-675* in fibroblast clones. qRT-PCR results were normalized to gene and miRNA expression in HepG2 cells.

The expression of *IGF2* in the SRShypo, SRSnormo, and Cnormo groups was below the detection limit of the quantitative real-time PCR (qRT-PCR) assay, whereas *IGF2* expression of the control HepG2 cell line was detected by the same assay (Figure 2a). In contrast, *H19* was expressed at higher, rather variable levels in all fibroblast clones, with no significant differences among the SRShypo, SRSnormo, and Cnormo groups (Figure 2a). The mean expression in the fibroblast clones was higher than that in the HepG2 cell line.

### Expression of *miR-483* and *miR-675* and their targets

The two miRNAs of the locus, *miR-483* and *miR-675*, were expressed at similar levels in all SRS fibroblast clones (Figure 2b). The expression of *miR-675* was significantly higher in the Cnormo group than in the SRShypo or SRSnormo group (*p* < 0.005), and *miR-483* showed a similar trend (*p* = 0.05; SRSnormo versus Cnormo). In the HepG2 control cell line, the amount of *miR-675* was comparable to that in the SRS fibroblast clones, though the concentration of *miR-483* was higher than in the SRS fibroblast clones.

The expression of the *miR-675* target *RB1* (retinoblastoma 1) was significantly increased in the SRShypo clones compared to the Cnormo clones (*p* = 0.007); a similar trend was observed for the expression of *IGF1R* (insulin-like growth factor 1 receptor) as well as for its ligand and *miR-483* target, *IGF1* (*p* = 0.043) (Additional file 1: Figure S1).

### IGF-II release into the supernatant

**Figure 3:**
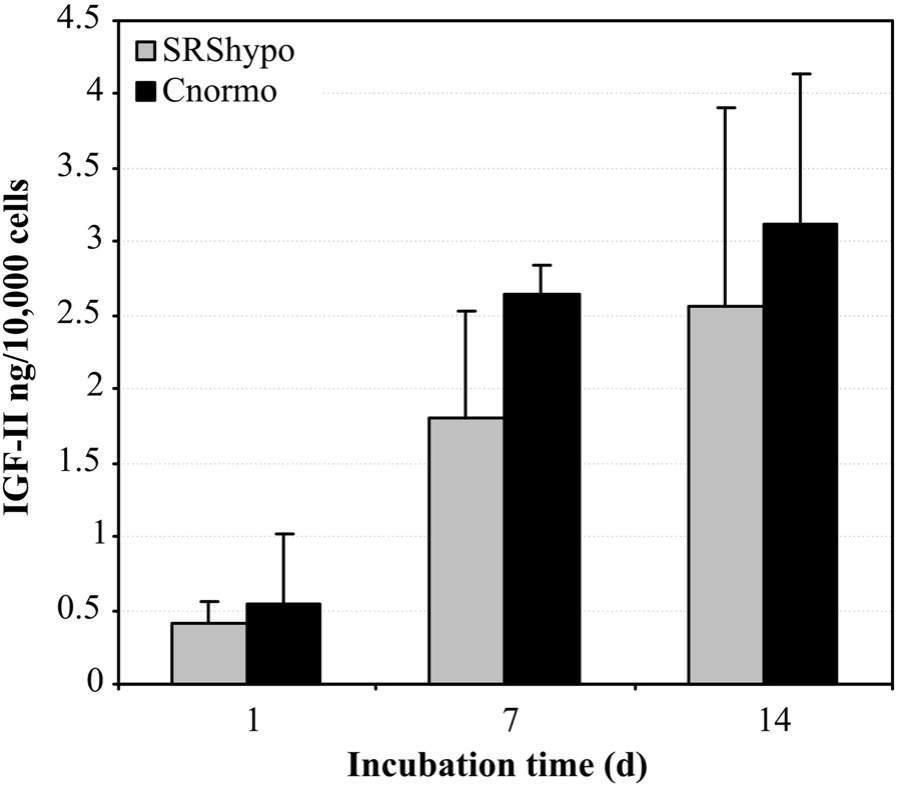
**IGF-II secretion by SRShypo und Cnormo clones.** SRShypo clones and Cnormo clones were cultured for 14 days (d) in standard medium. Cell count-based IGF-II secretion into the supernatant was measured on days 1, 7, and 14 using IGF-II RIA.

The cell number-normalized amount of the IGF-II protein measured in the supernatants after the incubation of 25,000 cells for 1, 7, and 14 days was not different in the SRShypo (S1sh_12, S5sh_11) and Cnormo clones (K3li_3, K3re_12) (Figure 3).

### Gene expression analysis

**Figure 4:**
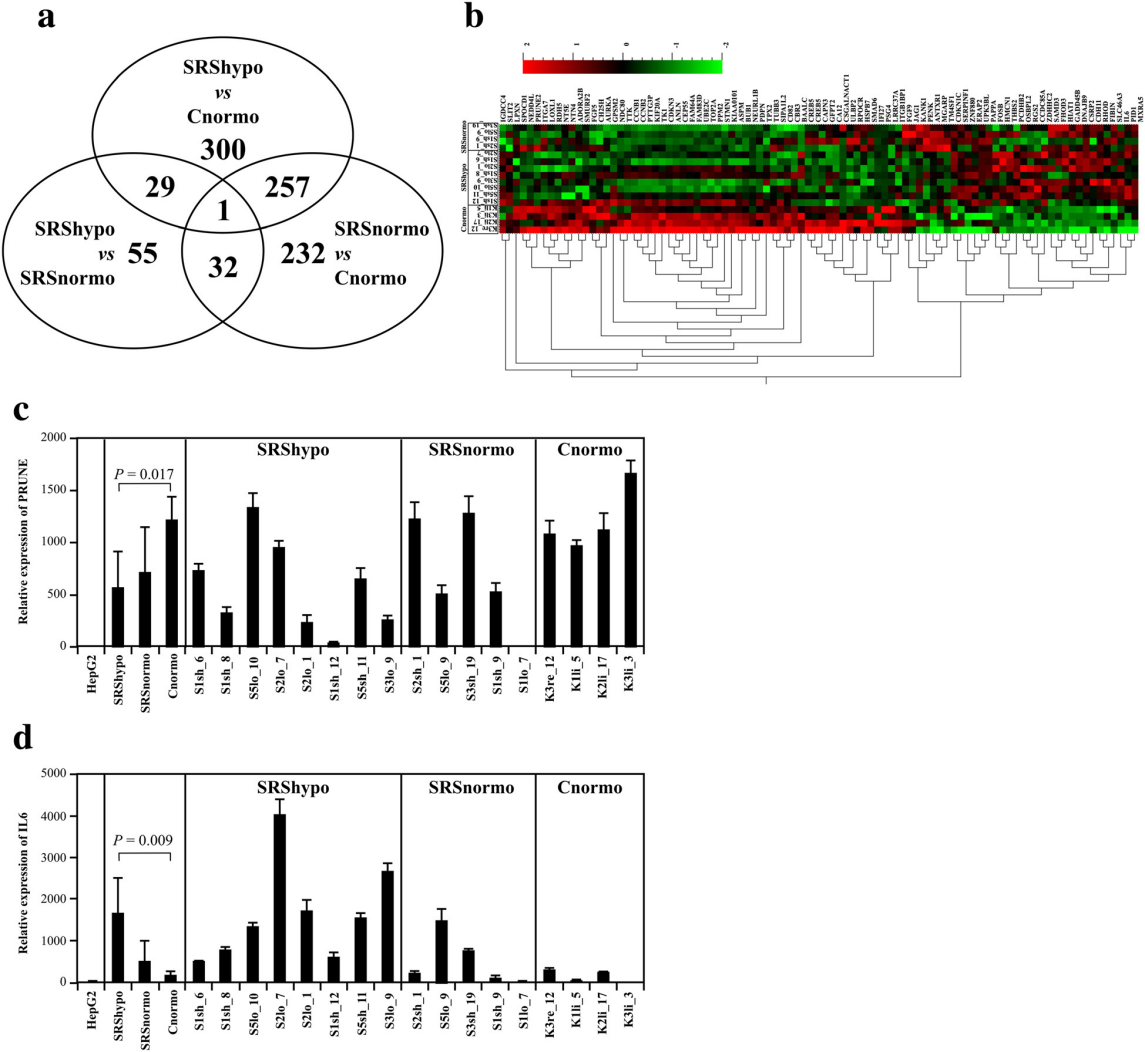
**Differential analysis results. (a)** Venn diagram of differentially regulated genes in the SRShypo, SRSnormo, and Cnormo clones. **(b)** Differentially regulated genes in the SRShypo, SRSnormo, and Cnormo groups. Clustering heatmap based on multigroup comparison using the statistical parameters *σ* = 0.174, *p* = 0.05, and *q* = 0.529103 in Qlucore Omics Explorer 3.0. Ninety-two genes were significantly differentially expressed between the three groups. The analysis of the SRSnormo clone S1lo_7 is not shown. **(c)** Differentially expressed *PRUNE2* in the SRShypo, SRSnormo, and Cnormo groups, as validated by qRT-PCR. qRT-PCR results were normalized to HepG2 cell gene expression. *PRUNE2* is expressed at a significantly lower level in SRShypo compared to Cnormo (*p* = 0.017). **(d)** Differentially expressed *IL6* in the SRShypo, SRSnormo, and Cnormo groups, as validated by qRT-PCR. qRT-PCR results were normalized to HepG2 cell gene expression. *IL6* is expressed at a significantly higher level in SRShypo compared to Cnormo (*p* = 0.009).

Microarray gene expression analyses were performed to profile differentially expressed genes. In total, 587 genes were found to be differently expressed in SRShypo compared to Cnormo, with 522 genes in SRSnormo compared to Cnormo (Figure 4a). The number of differentially expressed genes was significantly lower when comparing SRShypo and SRSnormo (*n* = 117). In the array, the expression of *IGF2* and *H19* was not significantly deregulated between groups of SRShypo, SRSnormo, and Cnormo.

A group comparison (SRShypo, SRSnormo, and Cnormo) of the differentially regulated genes is shown in a clustering heatmap (Figure 4b). For confirming the microarray results, *PRUNE2* (prune homolog 2) and *IL6* (interleukin 6) expression was validated by qRT-PCR (Figure 4c,d). *PRUNE2* expression was confirmed to be decreased in SRShypo (*p* = 0.017) but not in SRSnormo, and *IL6* expression was confirmed to be increased in SRShypo (*p* = 0.009) but was not significant in SRSnormo.

### Pathway analysis

Ingenuity Pathway Analysis (IPA) was performed on the expression changes between SRShypo and Cnormo and between SRSnormo and Cnormo. The canonical pathways that were negatively affected in the SRShypo and in the SRSnormo groups are shown in Additional file 2: Table S1. With respect to these pathways, the most highly activated upstream regulators identified by IPA were *TP53* (tumor protein p53), *CDKN1A* (cyclin-dependent kinase inhibitor 1A, p21), and *CDKN2A* (cyclin-dependent kinase inhibitor 1A, p16). As these pathways are involved in cell cycle regulation, the changes found were predicted to decrease cell proliferation and increase the delay of mitosis (Additional file 2: Table S1).

### Genes with the most severe change in expression

The genes at top of the list of the most differently expressed genes (FC > |3|) in SRShypo versus Cnormo and in SRSnormo versus Cnormo were identified. The expression of *SIPA1L2* (signal-induced proliferation-associated 1-like 2) and *NEDD4L* (neural precursor cell expressed, developmentally down-regulated 4-like) was significantly decreased in both types of SRS clones (*p* ≤ 0.026). In contrast, the expression of *GADD45B* (growth arrest and DNA-damage-inducible, beta), *PID1* (phosphotyrosine interaction domain containing 1), and *CSRP2* (cysteine and glycine-rich protein 2) was significantly increased in both SRS clones (*p* ≤ 0.022; Table 2).

**Table 2:** **Differentially expressed genes in SRShypo, SRSnormo, and Cnormo**

**Gene symbol**	**Gene title**	**RefSeq transcript ID**	**Column ID**	**SRShypo/Cnormo**		**SRSnormo/Cnormo**		**SRShypo/SRSnormo**	
				***p*** **value**	**FC**	***p*** **value**	**FC**	***p*** **value**	**FC**
SIPA1L2	Signal-induced proliferation-associated 1-like 2	NM_020808	11757924_s_at	0.017	−8.2	0.019	−10.7	0.738	1.3
NEDD4L	Neural precursor cell expressed, developmentally down-regulated 4-like	NM_001144964 NM_001144965 NM_001144966 NM_001144967 NM_001144968	11722423_a_at	0.015	−5.0	0.026	−5.3	0.936	1.0
PROCR	Protein C receptor, endothelial	NM_006404	11737798_a_at	0.011	−3.0	0.925	1.0	0.009	−3.1
NR4A2	Nuclear receptor subfamily 4, group A, member 2	NM_006186	11725632_at	0.034	3.3	0.943	1.0	0.040	3.2
FOSB	FBJ murine osteosarcoma viral oncogene homolog B	NM_001114171 NM_006732	11717345_a_at	0.029	3.9	0.983	−1.0	0.027	4.0
GADD45B	Growth arrest and DNA-damage-inducible, beta	NM_015675	11757865_a_at	0.001	4.2	0.007	3.4	0.535	1.2
CSRP2	Cysteine and glycine-rich protein 2	NM_001321	11729239_x_at	0.001	4.3	0.001	4.6	0.869	−1.1
PID1	Phosphotyrosine interaction domain containing 1	NM_001100818 NM_017933	11721983_a_at	0.005	4.6	0.022	3.9	0.706	1.2
CCDC85A	Coiled-coil domain containing 85A	NM_001080433	11744318_at	0.005	5.4	0.977	1.0	0.005	5.3

*FC* fold change.

In addition, SRShypo gene expression was compared to that of SRSnormo (FC > |3|), and *PROCR* (protein C receptor, endothelial) was the only gene found to be downregulated in SRShypo compared to SRSnormo (*p* = 0.009). The expression of *CCDC85A* (coiled-coil domain containing 85A), *FOSB* (FBJ murine osteosarcoma viral oncogene homolog B), and *NR4A2* (nuclear receptor subfamily 4, group A, member 2) was all upregulated in the SRShypo group (*p* ≤ 0.04; Table 2).

## Discussion

In most of the known human imprinting disorders, the biological link between the epigenetic mutation and its biological and clinical manifestation remains missing. Indeed, the significant progress in the understanding of genetics and of clinical characteristics has not yet contributed to bridging the gap between the areas of genotyping and of phenotyping in imprinting disorders.

Hypomethylation of the paternal *ICR1* of the *IGF2/H19* locus on chromosome 11p15 is the most frequent finding observed in SRS. It is thought that this hypomethylation enables the binding of the insulator protein CTCF to the paternal allele. The physiological binding of CTCF to the maternal allele suppresses *IGF2* expression and promotes *H19* expression via the relocation of enhancer elements. Therefore, hypomethylation of the paternal allele is likely to introduce the same expression regulation to the paternal genome and, consequently, a decrease in *IGF2* expression and an increase in *H19* expression [23,24].

However, in our previous *in vitro* study on skin fibroblasts from children with SRS, this change in gene expression was not observed [2]. This discrepancy between theory and the results obtained could have been caused by the mosaic distribution of hypomethylated cells in tissues from children with SRS and, particularly, by the comparatively low degree of hypomethylation in our skin fibroblast cultures [2]. Indeed, the cellular dysfunction of cells with hypomethylation could be masked by the presence of a majority of normomethylated cells in the same culture.

Aiming to overcome this problem, we selected single cells and established clonal cultures of skin fibroblasts from children with SRS. The methylation analysis confirmed that the technique employed enabled the establishment of cell lines with severe *ICR1* hypomethylation as well as with normomethylation. Furthermore, the degree of methylation did not change during passaging. In contrast to our hypothesis, IGF-II secretion and *H19* expression did not reveal any difference between the hypomethylated and the normomethylated groups of SRS skin fibroblast clones, in concordance with the results from non-clonal fibroblast cultures in our previous study [2]. Although IGF-II was detected by radioimmunoassay (RIA) in the supernatant of hypomethylated SRS clones and control clones at a comparable level after 7 days of culture, our qRT-PCR assay was not sensitive enough to reliably measure the very low amounts of *IGF2* mRNA in these clones in comparison to a housekeeping gene.

In a very recent paper, Azzi et al. [25] reported on *IGF2* expression in six non-clonal hypomethylated and one normomethylated skin fibroblast cell lines from patients with SRS. Although they found a relationship between *IGF2* expression and methylation status within the group of SRS fibroblasts with a methylation index ranging from 0% to 45%, *IGF2* expression in SRS fibroblasts was not significantly different from the control fibroblasts. This finding implies that the gene expression changes found were of minor severity; thus, the biological meaning is still unclear.

The expression of the miRNAs *miR-483* and *miR-675*, both expressed from the *IGF2/H19* locus, and of their potential targets varied between the SRS and control clones. In agreement with the expression of *IGF2* and *H19*, *miR-483* was expressed at a lower level than *miR-675* compared to the HepG2 cell line. Overall, our *in vitro* data do not support the notion of a major role of these miRNAs in the etiology of SRS.

One can speculate that the relevant biological mechanisms in SRS linked to the *IGF2/H19* locus are developmental stage-dependent and not traceable after birth. Within this context, the presence of the so-called multilocus methylation defects shown in 7% of SRS patients [5] or more [22,25] may have to be considered. In a previous study, multilocus methylation defects outside the 11p15 locus were found in two out of the four SRS patients reported here [22]. The role of these additional methylation defects outside the IGF2/H19 locus in the pathogenesis of SRS is still undefined and needs further investigations. Our actual approach was not designed to estimate the effects of *multilocus* methylation defects.

After excluding a clear effect on gene expression of the *ICR1* hypomethylated *IGF2/H19* locus in SRS clones, we aimed to characterize the total expression of these fibroblast clones by microarray expression profiling. The gene expression profiles of the SRS and control clones were clearly different. We observed a greater degree of similarity between the two groups of SRS clones (hypomethylated versus normomethylated) than between each of these groups and the group of control clones derived from healthy patients. Interestingly, the pathway analysis of the SRS clones showed the aberrant expression of genes involved in cell proliferation, cell cycle control, and timing of mitosis. The data implicate a decreased proliferation of cells and an increased delay in mitosis in both SRShypo and SRSnormo fibroblast clones. In line with this genetic observations, our *in vitro* studies on these fibroblast clones revealed a slower cell proliferation of the tested *ICR1* hypomethylated SRS clones compared to normomethylated control clones.

Specifically, genes of the “mitotic roles of polo-like kinase” pathway were exclusively downregulated in both the SRShypo and SRSnormo clones, genes of the FAK (focal adhesion kinase) pathway were downregulated only in the SRShypo clones, and genes of the pathways “cell cycle: G2/M DNA damage checkpoint regulation” and “cell cycle control of chromosomal replication” were downregulated in the SRSnormo clones. These global pathway changes were associated with the increased expression of genes in the signaling cascades of the upstream regulators *TP53* and *CDKN1A* in SRS clones. *GADD45B* was more highly expressed in the SRS clones than in the controls. The encoded protein belongs to the p53-regulated downstream cascade, and its upregulation was found to arrest primary human fibroblasts at G2/M [26].

One limitation of our analysis is the lack of proliferation studies and studies on the IGF-II release from *normomethylated* SRS clones. These experiments were beyond the scope of our actual study. A second limitation is that patients and controls were not age- and sex-matched.

## Conclusions

In conclusion, a group of severely *ICR1* hypomethylated fibroblasts derived from single cells did not reveal differences from a group of *ICR1* normomethylated fibroblasts with respect to IGF-II secretion and to *IGF2*, *H19,* and *miR-483/675* expression. Interestingly, the normomethylated control clones proliferated faster than the *ICR1* hypomethylated SRS clones. These functional findings were corroborated by gene expression changes in SRS cell clones, predicting decreased cell proliferation and delayed mitosis. Thus, a more complex network of genes might be involved in SRS-associated growth impairment than assumed thus far.

## Methods

### Patients

The protocol of this study was approved by the Ethics Committee of the Medical Faculty of Tuebingen. All SRS patients, healthy volunteers, and their parents provided written informed consent. The SRS patients were recruited in our endocrine clinic.

The mean age of the SRS patients (S1–S5) was 13.4 years (range 9.3–16.6) and that of the controls (K1–K3) was 14.6 years (range 14.0–15.0) [2] (Table 3). The three healthy controls were all male, aged 14, 14.7, and 15 years. All four SRS patients (three females) had severe intrauterine growth retardation (birth weight range −5.68 to −3.46 standard deviation score (SDS)), a triangular face, body asymmetry, and relative macrocephaly (Table 3). Growth hormone (GH) therapy was performed in patients S2, S3, and S5 during this study. Previous analysis revealed methylation defects outside of the 11p15 locus in patients S1 and S3 [22]. The control individuals were recruited in our Pediatric Surgery Department where they underwent anesthesia for the removal of metals after arm fracture healing.

**Table 3:** **Clinical characteristics of the patients with Silver-Russell syndrome and 11p15** ***ICR1*** **hypomethylation**

		**Patient**			
		**S1** ^**a**^	**S2**	**S3** ^**a**^	**S5**
Sex [M/F]		M	F	F	M
Duration of gestation [weeks]		40	39	39	39
Birth weight	[g]	1,560	1,380	1,960	1,800
	[SDS]	−5.7	−5.4	−3.5	−4.2
Birth length	[cm]	40	38	45	44
	[SDS]	−6.0	−6.1	−2.4	−3.2
Age [years]		15.0	16.6	9.2	16.3
Height	[cm]	149.1	151.7	129.7	151.5
	[SDS]	−2.6	−2.2	−0.9	−3.4
Weight [kg]		30.1	27.1	27.2	61.0
BMI [kg/m^2^]		13.5	11.8	16.2	26.6
Pubertal stage [Tanner stages]		G4PH4	B5PH5	B1PH2	G5PH5
Normal cognitive development		+	+	+	+
Asymmetry		+	+	+	+
Triangular face		+	+	+	+
Bossing forehead		+	+	+	+
GH therapy		+	+	+	-

^a^Two patients with additional methylation defects outside the 11p15 locus.

### Establishment of clonal fibroblast cultures and cultivation of HepG2 cells

Skin biopsies of both arms were obtained from SRS patients (*n* = 4) and healthy volunteers (*n* = 3), as previously described [22]. Primary fibroblast cultures (FC) were cultivated in RPMI (Invitrogen, Karlsruhe, Germany) supplemented with 10% fetal calf serum (FCS; Biochrom, Berlin, Germany) and 100 units/ml penicillin/streptomycin (Biochrom) and incubated in a 37°C humidified atmosphere containing 5% CO_2_.

Under microscopic control, single cells were picked from trypsinized FC after passages 4–8 and seeded into 96-well plates. Cell-free medium from the same well served as the negative control. The clonal fibroblasts were expanded and yielded suitable amounts of cells at passages 12–18. The hepatocellular carcinoma cell line HepG2, which is commonly implemented in studies of *IGF2* signaling [19,27], was used as a technical control and cultured under the abovementioned standard conditions. The nomenclature of the cell clones refers to the patient or the control (S = SRS patient, K = control); to the arm where the skin biopsy was taken, in the case of SRS patients to the length of the arm (sh = short side, lo = long side) and in the case of the healthy controls to the site of the arm (li = left side, re = right side); and to the number of the specific clone.

### Isolation of nucleic acids (DNA, RNA, miRNA)

DNA, RNA, and miRNA were isolated from clonal fibroblasts and HepG2 cells using the DNeasy Mini Kit or miRNeasy Mini Kit (Qiagen, Hilden, Germany), respectively. The concentrations of the nucleic acids were determined using a Nanodrop spectrophotometer (Thermo Scientific, Wilmington, DE, USA).

### Methylation analysis

The methylation status of *ICR1* in the *IGF2/H19* locus (*H19DMR*) and of *ICR2* in the *KCNQ1* gene (*KvDMR*) was measured with MS-MLPA using the Salsa MS-MLPA kit ME030-C1 (MRC-Holland, Amsterdam, The Netherlands) [28]. In brief, DNA was digested with a DNA methylation-sensitive enzyme (*Hha*I) specific for the CpG-containing sequence GCGC. Protected methylated DNA was subsequently ligated and PCR amplified. For each of the four investigated CpG sites (M1–4), a PCR product of a specific length surrounding the site was obtained. For each reaction, 200 ng of genomic DNA was used. The extent of methylation at a specific site (expressed in %) was calculated from reference PCR reactions for genomic sequences lacking a GCGC tetranucleotide and from a parallel reaction of the same, but undigested, genomic DNA sample.

MS-MLPA results for *ICR1* methylation were validated by bisulfite sequencing. Bisulfite conversion of fibroblast-derived DNA from two clones (S1sh_8 and S5sh_11) was performed using the EZ DNA Methylation-Gold™ Kit (Zymo Research, Irvine, CA, USA). Primers flanking the methylation sites M1–4 in *ICR1* were designed complementary to the bisulfite-converted sequence (SRSBSfor3, 5′-TTATGGGAATAGAGGGTTTG-′3, and SRSBSrev1, 5′-CCACTATCTCCCCTCAA-′3). The PCR conditions consisted of an initial period of denaturation at 95°C for 5 min, followed by 40 cycles of 30 s of denaturation at 95°C, 30 s of annealing at 57°C, and 1 min of extension at 72°C, and a final period of extension at 72°C for 6 min. Gel electrophoretic analysis of the PCR product revealed a band of 712 base pairs. The PCR products were subcloned into pGEM-T easy (Promega, Mannheim, Germany) and sequenced by Sanger sequencing (GATC, Konstanz, Germany).

### Microarray analysis

After RNA extraction from fibroblast clones in the exponential growth phase of similar passages (P12–18), microarray gene expression analyses were performed using Affymetrix GeneChip HT HG-U219 PM 96-array plates at the Microarray Facility Tuebingen (MTF Services, Medical Genetics, Tuebingen, Germany). This array measures the expression of more than 36,000 transcripts and variants, representing more than 20,000 genes mapped through UniGene or via RefSeq v36 annotation. For this purpose, hypomethylated clones from patients (<10%, named SRShypo) and normomethylated clones from patients (30%–55%; named SRSnormo) as well as control fibroblasts (named Cnormo) were selected. For the analysis, 250 ng of total RNA was processed with the GeneChip IVT Express Kit (Part no. 901229) and hybridized to the GeneChip Human Genome U219 array plate with specific protocols for the use of GeneTitan Hybridization, Wash and Stain Kit for IVT Array Plates (P/N 901530). A GeneTitan instrument was used to hybridize, wash, stain, and scan the arrays. Affymetrix GeneChip Command Console 3.1 was used to control the process and to summarize the probe cell intensity data. The hybridization quality was checked with Affymetrix GeneChip Command Console and Expression Console™ 1.1 s.

Files containing a single intensity value for each probe region delineated by a grid on each array image were imported into Partek Genomics Suite (version 6.6, Partek, St. Louis, MO, USA) for probe set summarization and statistical analysis. A model based on Robust Multichip Analysis was performed as the method for probe set summarization to obtain a single intensity value representing the transcript abundance for each probe set and, thus, to enable comparisons between arrays by normalizing and logarithmically transforming the array data and stabilizing the variance across the arrays.

### cDNA synthesis and qRT-PCR analyses and gene and miRNA expression analyses

Reverse transcription of total RNA was performed using the miScript RT PCR Kit (Qiagen). Mimics (*miR-483*-3p and *miR-675*-5p) used as standards for absolute quantification were reversely transcribed following the manufacturer’s instructions (Qiagen). For the determination of gene expression, qRT-PCR was performed with 2.5 ng reversely transcribed total RNA per sample. Glyceraldehyde 3-phosphate dehydrogenase (*GAPDH*) and *SNORD72* were used as the reference for cDNA normalization. The qRT-PCR reactions were performed with the following primers: *IGF2*for, 5′-CAGTGAGACCCTGTGCGGCG-′3; *IGF2*rev, 5′-TCCCTCTCGGACTTGGCGGG-′3; *H19*for, 5′-TGAGCTCTCAGGAGGGAGGATGGT-′3; *H19*rev, 5′-TTGTCACGTCCACCGGACCTG-′3; *GAPDH*for, 5′-TGCACCACCAACTGCTTAGC-′3; *GAPDH*rev, 5′-GGCATGGACTGTGGTCATGAG-′3. QuantiTect Primer Assays (*IGF1, IGF1R, RB, IL6, PRUNE*), miScript Primer Assays (*miR-483*-3p, *miR-675*-5p), and the SYBR Green PCR Kit (Qiagen) were utilized. The amplifications were carried out in triplicate using a CFX96 cycler (Bio-Rad, Munich, Germany) with the following thermocycling conditions: 95°C for 15 min, followed by 40 cycles of 15 s at 94°C, 15 s at 55°C, and 30 s at 70°C for Primer Assays; or 95°C for 5 min, followed by 40 cycles of 15 s at 95°C, 15 s at 60°C, and 60 s at 72°C for the *IGF2*, *H19*, and *GAPDH* primers. Gene and miRNA expression was quantified relative to *GAPDH* or *SNORD72*, respectively, using the ddCt method.

### Cell proliferation assay and IGF-II RIA

In 12-well plates, 2.5 × 10^3^ clonal fibroblasts were cultured under standard conditions. After 1 (d1), 7 (d7), and 14 days (d14), proliferation of the plated cells was measured using MTT (Sigma-Aldrich). Briefly, plated clonal fibroblasts were incubated with 100 μl MTT for 4 h at 37°C, and 100 μl per well of 10% sodium dodecyl sulfate solution in 0.01 M hydrochloric acid was added. After overnight incubation at 37°C, the absorbance of the solubilized formazan crystals was measured (Victor, Perkin Elmer, USA). The mean values of two to three wells per culture were determined, and the experiments were performed with four different SRShypo clones (S1sh_12, S2lo_7, S2lo_1, S5sh_11) and three different Cnormo clones (K2li_17, K3li_3, K3re_12).

After 1 (d1), 7 (d7), and 14 days (d14) of culture, supernatants were collected to quantify IGF-II production and secretion by the clonal fibroblasts using RIA [2]. The mean values of three wells per culture were determined, and the experiments were performed with two different SRShypo clones (S1sh_12, S5sh_11) and two different Cnormo clones (K3li_3, K3re_12). The IGF-II amount measured was corrected for the cell number by extrapolation using a standard curve.

### Statistical analyses

The data are presented as the mean and standard deviation. The data from the qRT-PCR, protein, and proliferation assays were compared using the two-sided unpaired *t* test, and the microarray data were analyzed using ANOVA. A *p* value was calculated by evaluating the significance of the difference observed in the mean transcript abundance for each probe set between the SRShypo, SRSnormo, and Cnormo groups. When necessary, the *p* values were corrected for multiple testing with the 5% FDR-based method of Benjamini and Hochberg [29]. A *p* value below 0.05 was considered statistically significant. The probe sets exhibited significant, greater than 1.5-fold, differences in mean transcript abundance among the described cultures. A hierarchical cluster analysis was performed with Qlucore Omics Explorer 3.0 (Qlucore AB, Lund, Sweden). Possible biological pathways and the inter-relationships between network genes in the subsets of candidate genes that had particularly interesting patterns were analyzed using IPA® (QIAGEN). The microarray data have been deposited at the NCBI Gene Expression Omnibus (accession number GSE61120).

## Additional files

Additional file 1: Figure S1. Differential expression of the *miR-675* targets *RB1* and *IGF1R* and the *miR-483* target *IGF1* in SRShypo, SRSnormo, and Cnormo groups. qRT-PCR results were normalized to HepG2 cell gene expression. *RB1* and *IGF1* are expressed at a significantly higher level in SRShypo compared to Cnormo (*p* ≤ 0.043). Additional file 2: Table S1. Ingenuity Pathway Analysis. The differentially regulated pathways, upstream regulators, and functions are shown. The data comprise the comparisons of SRShypo versus Cnormo and of SRSnormo versus Cnormo. **p* < 0.001; ↓ All genes in this pathway are downregulated.
