# Supplementary figures and images for: Decreased expression of cell proliferation-related genes in clonally derived skin fibroblasts from children with Silver-Russell syndrome is independent of the degree of 11p15 ICR1 hypomethylation
Source: Clin Epigenetics. 2015 Jan 22;7(1):5. doi: 10.1186/s13148-014-0038-0 (PMC4318184; doi:10.1186/s13148-014-0038-0)

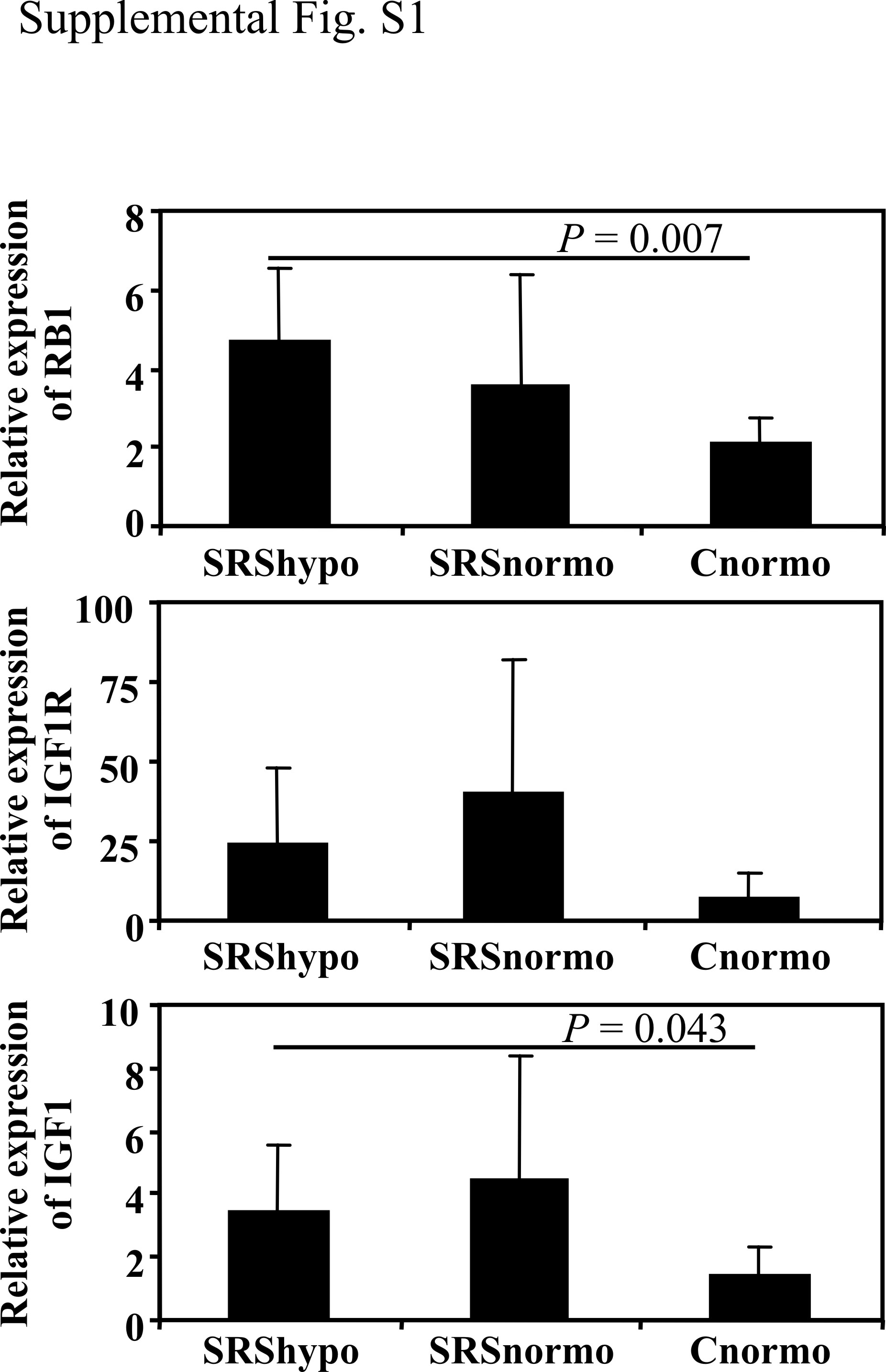

Supplement: Additional file 1: Figure S1. — Differential expression of the miR-675 targets RB1 and IGF1R and the miR-483 target IGF1 in SRShypo, SRSnormo, and Cnormo groups. qRT-PCR results were normalized to HepG2 cell gene expression. RB1 and IGF1 are expressed at a significantly higher level in SRShypo compared to Cnormo (p ≤ 0.043). [file 13148_2014_38_MOESM1_ESM.tiff]
