# Supplementary material for: Decreased expression of cell proliferation-related genes in clonally derived skin fibroblasts from children with Silver-Russell syndrome is independent of the degree of 11p15 ICR1 hypomethylation
Source: Clin Epigenetics. 2015 Jan 22;7(1):5. doi: 10.1186/s13148-014-0038-0 (PMC4318184; doi:10.1186/s13148-014-0038-0)
Supplement: Additional file 2: Table S1. — Ingenuity Pathway Analysis. The differentially regulated pathways, upstream regulators, and functions are shown. The data comprise the comparisons of SRShypo versus Cnormo and of SRSnormo versus Cnormo. *p < 0.001; ↓ All genes in this pathway are downregulated. [file 13148_2014_38_MOESM2_ESM.docx]

**Table S1**

**Ingenuity Pathway Analysis: the differentially regulated pathways, upstream regulators and functions** **are shown.**

The data comprise the comparisons of SRShypo versus Cnormo and of SRSnormo versus Cnormo. * p < .001; ↓ All genes in this pathway are downregulated.
